# Pesticide Exposure and Breast Cancer: Molecular Mechanisms, Epidemiological Evidence, and Current Challenges

**DOI:** 10.3390/jox16060192

**Published:** 2026-10-09

**Authors:** Emine Tasan, Giuseppina Augimeri, Daniela Bonofiglio, Giuseppina Peluso, Stefania Marsico, Adele Vivacqua, Pietro Rizza, Saveria Aquila

**Affiliations:** 1 Department of Pharmacy and Health and Nutrition Sciences, University of Calabria, 87036 Arcavacata di Rende, Italy; emine.tasan@unical.it (E.T.); giuseppina.augimeri@unical.it (G.A.); daniela.bonofiglio@unical.it (D.B.); stefania.marsico@unical.it (S.M.); pietro.rizza@unical.it (P.R.); saveria.aquila@unical.it (S.A.); 2 Health Center, University of Calabria, 87036 Arcavacata di Rende, Italy; 3 Sperm Bank, Department of Maternal Infant, Annunziata Hospital, 87100 Cosenza, Italy; g.peluso@aocs.it

**Keywords:** breast cancer, pesticides, endocrine-disrupting chemicals, estrogen receptors, GPER, aromatase, oxidative stress, tumor progression, therapeutic resistance, phytochemical compounds

## Abstract

Breast cancer is a heterogeneous disease driven by complex interactions between genetic, hormonal, metabolic, lifestyle, and environmental factors. Among environmental exposures, agricultural pesticides represent potential contributors to breast carcinogenesis through endocrine-disrupting, oxidative, genotoxic, and epigenetic mechanisms. Utilizing a structured search strategy across PubMed, Scopus, and Web of Science, this review provides a critical synthesis of current evidence linking major pesticide classes with breast cancer dynamics, strictly distinguishing preclinical mechanistic findings from human epidemiological data. Special attention is given to endocrine-disrupting pathways involving estrogen receptors and aromatase hyper-induction, alongside oxidative stress, matrix metalloproteinase activation, epithelial–mesenchymal transition, cancer stemness expansion, and therapeutic resistance. We further evaluate the cytoprotective potential and severe bioavailability bottlenecks of dietary phytochemicals in mitigating pesticide-induced damage. Finally, we address critical methodological limitations, such as exposure misclassification, confounding factors, and the gap between micromolar in vitro doses and realistic nanomolar human exposures, stressing that preclinical mechanisms cannot prove direct clinical causality Therefore, integrating prospective multi-omics biomonitoring with cumulative cocktail risk assessments is essential for evidence-based primary prevention.

## 1. Introduction

Breast cancer is the most frequently diagnosed neoplastic disease and remains the leading cause of cancer mortality among women worldwide [1]. According to recent global epidemiological analyses, the incidence of this cancer is rising steadily, with over 2.3 million new cases diagnosed each year, making this disease a key public health priority [2]. Histologically, breast cancer is a highly heterogeneous malignant disease originating from the epithelial component of the mammary gland, characterized by a complex diversity in its etiopathogenesis, clinical subtypes, and underlying pathophysiology [3]. Accurate determination of the histological and molecular characteristics of breast cancer has become essential for developing and tailoring treatment strategies, ranging from endocrine therapies to new, biomarker-based, patient-specific targeted therapies [4]. While early diagnosis and adherence to population screening programs yield an excellent prognosis when the malignancy is localized strictly to the breast, achieving a five-year relative survival rate of nearly 99%, the overall global mortality rate remains a significant challenge [2,5]. Clinical data confirm that chemotherapy and radiotherapy remain the foundational pillars of standard treatment; however, their long-term effectiveness is often compromised by cancer cells developing resistance following prolonged exposure [6]. This clinical hurdle has driven intensive scientific research over the last few years to unravel the molecular mechanisms driving therapeutic evasion [7,8,9,10].

The complex etiology of breast cancer is mirrored in its molecular classification, which dictates not only the clinical behavior of the disease, but also its personalized therapeutic response [3]. Breast cancer is broadly categorized into four primary molecular subtypes based on receptor expression and proliferation indexes.

The risk factors driving the development of these molecular subtypes are multifaceted and deeply interconnected, spanning genetic, hormonal, reproductive, and lifestyle variables. The hereditary and genetic component contribute to approximately 5% to 10% of total cases, primarily driven by pathogenic germline mutations in the tumor suppressor breast cancer (BRCA)1 and BRCA2 [11]. Consequently, women with a family history of breast cancer, particularly amongst first-degree relatives, are exposed to a significantly higher risk than the general population [12]. Alongside genetics, hormonal exposure plays a pivotal role, particularly due to the stimulating effect of estrogen on the hormone receptor-positive luminal tumors. Estrogens exert their carcinogenic potential via two main pathways: active signaling mediated by the classic oestrogen receptors (ER) α and β and by the G-protein-coupled estrogen receptor (GPER), which alters cellular gene expression; and the oxidative metabolism of estrogens themselves, which generates reactive species capable of causing direct damage to DNA [13]. Accordingly, the duration of a woman’s cumulative lifetime exposure to estrogens linearly influences her risk profile. Reproductive history highlights that early menarche exposes women to hormones longer, raising their susceptibility, whereas a full-term pregnancy and longer durations of breastfeeding appear to exert protective, modulating effects on the mammary epithelium [1,12,14]. Hormonal regulation is also heavily modulated by modifiable lifestyle factors, among which obesity is particularly prominent. In postmenopausal women, adipose tissue becomes the primary site of estrogen synthesis via aromatase activity, explaining the direct correlation between excess body weight and the onset of hormone receptor-positive tumors [15]. Although obesity presents a striking biological paradox, correlating with a slightly lower risk of breast cancer onset in premenopausal women while significantly elevating risk in postmenopausal cohorts, it acts as a universally adverse prognostic factor once the disease is established. Regardless of age or menopausal status, excess body weight is systematically associated with a more advanced clinical stage at diagnosis and a poorer overall prognosis. This ubiquitous decline in survival is driven by distinct yet overlapping pathophysiological mechanisms, ranging from localized adipose-derived hyperestrogenism in postmenopausal women to systemic chronic inflammation, insulin resistance, and altered adipokine signaling in premenopausal patients [16]. Interestingly, recent evidence suggests that susceptibility to breast cancer can be programmed during the earliest stages of life, where maternal diet and early-life nutritional status affect long-term breast cancer risk [17]. Conversely, regular physical activity serves as a strong protective factor for breast cancer onset, lowering risk by reducing systemic inflammation and circulating hormone levels [18]. Among harmful voluntary habits, chronic alcohol consumption and tobacco smoking show a clear dose–response relationship with increased disease susceptibility, driven by both systemic toxicity and systemic endocrine disruption [12,19].

The primary clinical challenge remains the progression of the disease toward its terminal stages, a trajectory frequently driven by the acquisition of drug resistance that enhances the invasive and migratory potential of cancer cells [6]. The metastatic cascade, wherein cancer cells disseminate from the primary tumor to surrounding tissues and distant organs, is the leading cause of cancer morbidity and is responsible for approximately 90% of breast cancer-related deaths [20]. It is precisely the limitation of conventional treatments in thwarting resistant and metastatic forms that has prompted the scientific community to shift its focus toward identifying new paradigms in primary prevention. In this context, while endogenous risk factors and determined lifestyle choices account for a significant portion of pathogenesis, growing attention must be directed toward the impact of inevitable environmental contaminants.

Everyday exposure to synthetic chemical formulations represents a critical yet under-evaluated variable. Plastic food and beverage containers, cosmetics, sunscreen, cleaning products and garden products all contain chemicals. These compounds, which frequently contaminate the food supply and groundwater, are no longer viewed merely as generic toxins, but as active biological agents capable of deeply altering human physiology [21].

Among these environmental factors, a prominent role is played by agricultural pesticides [22,23]. Acting as potent endocrine-disrupting chemicals (EDCs), these agents can directly interfere with the normal synthesis, metabolism, and cellular signaling of the endocrine system, mimicking or blocking natural hormones [24].

Exposure to these substances, especially during vulnerable periods of development and increased susceptibility, is thought to contribute to the development of breast cancer and reduce the efficacy of hormone therapy. Given their widespread presence and their bioaccumulative nature, it is essential to understand the potential carcinogenicity of these contaminants and their specific signalling pathways. In this context, by distinguishing between preclinical molecular observations and epidemiological associations in humans, we aim to provide a nuanced overview of current evidence, refraining from broad generalizations and focusing instead on distinct, representative pesticide classes and their specific molecular mechanisms.

## 2. Search Strategy and Literature Selection Criteria

To ensure a rigorous and comprehensive synthesis of the available evidence, a structured search strategy was applied across PubMed/MEDLINE, Scopus, and Web of Science databases for articles published up to July 2026. The search algorithm incorporated combinations of Medical Subject Headings (MeSH) and free-text terms relevant to three main domains: exposure: (“pesticides” OR “herbicides” OR “insecticides” OR “fungicides” OR “glyphosate” OR “atrazine” OR “chlorpyrifos” OR “p,p′-DDT” OR “p,p′-DDE” OR “mancozeb”); pathology/mechanisms: (“breast cancer” OR “mammary gland” OR “endocrine disruption” OR “estrogen receptor” OR “GPER” OR “aromatase” OR “oxidative stress” OR “epithelial-mesenchymal transition” OR “cancer stem cells”); mitigation: (“phytochemicals” OR “nutraceuticals” OR “Nrf2” OR “detoxification enzymes”). Inclusion criteria encompassed: original in vitro, in vivo, and translational research investigating the molecular mechanisms linking pesticide exposure to breast cancer initiation, progression, or therapeutic resistance; prospective and retrospective epidemiological or biomonitoring studies assessing human internal exposure and breast cancer risk; studies evaluating dietary phytochemicals against pesticide-induced cell damage. Exclusion criteria included non-English articles, conference abstracts without full text, studies lacking clear biological or toxicological relevance to breast tissue, and studies evaluating unrelated chemical contaminants. Reference lists of eligible studies and relevant review articles were manually screened to capture additional pertinent literature.

## 3. Pesticide Exposure and Mammary Bioaccumulation

The term “pesticide” represents a broad, chemically diverse classification of synthetic and organic compounds formulated to repel, destroy, or mitigate pests. In particular, pesticides represent an extremely heterogeneous spectrum of chemical entities characterized by vastly divergent physicochemical properties, toxicokinetics, persistence, and modes of action. Consequently, biological outcomes and tissue distribution patterns cannot be uniformly generalized across all agrochemicals. To accurately evaluate their individual contributions to breast tissue disruption, three distinct toxicokinetic profiles must be recognized:

- Persistent and bioaccumulative compounds (highly lipophilic compounds, such as legacy organochlorines), which are highly persistent in the environment, resist biodegradation and bioaccumulate over decades within the lipid-rich adipose stroma of the human mammary gland.
- Transient Parent Compounds (e.g., modern organophosphates, triazines, carbamates), hydrophilic or moderately lipophilic non-persistent pesticides that have short biological half-lives. They circulate primarily in blood plasma, undergoing rapid hepatic phase I/II metabolism and clearance without undergoing long-term adipose sequestration.
- Metabolites as Exposure Biomarkers that are stable metabolic clearance products excreted in urine or systemic fluids. They reflect recent, ongoing environmental exposure rather than long-term tissue bioaccumulation.

Of note, in real-world scenarios, human populations are rarely exposed to these chemical classes in isolation. Instead, individuals are exposed to complex, low-dose pesticide mixtures (‘cocktail effect’) that can exert cumulative, additive, or multi-target biological effects within the body. Moreover, pesticides are rarely confined to their initial application sites. Because of their physical-chemical properties, such as volatility, water solubility, and environmental persistence, they cycle continuously through natural and built environments. Generally, dietary intake is the most persistent and significant route of chronic, low-dose pesticide exposure. These residues often persist through industrial food processing, appearing in consumer staples such as fruits, vegetables, grains, and processed foods. This dietary exposure is further compounded by water contamination. Pesticides applied to crops can leach vertically through soil profiles or be carried by surface runoff into rivers, lakes, and shallow aquifers. Soluble herbicides and their stable environmental metabolites are highly mobile in water systems [25]. Because standard municipal drinking water plants are rarely equipped to completely eliminate these low-molecular-weight organic micropollutants, trace levels of multiple pesticides are frequently detected in tap and bottled drinking water, representing a source of daily, low-dose ingestion [26].

For populations living near agricultural basins, inhalation and dermal contact present significant exposure risks. During pesticide applications, a substantial portion of the active ingredient can drift away from the target area as fine aerosols, a process known as agricultural spray drift [27]. These airborne chemical droplets can be carried by wind currents into nearby residential areas, where they are inhaled or absorbed through the skin.

The clinical relevance of these exposure pathways is demonstrated by the systemic internalization and internal burden of pesticides within the human body. Large-scale biomonitoring studies consistently detect multiple pesticide residues and their metabolites in human biological fluids. Urine analyses reveal stable metabolic products indicating continuous short-term exposures, whereas blood serum tests and adipose tissue biopsies confirm that a vast majority of the population in industrialized and agricultural countries harbors a measurable body burden of persistent, bioaccumulate organochlorines [26,28].

From the perspective of breast oncology, the most critical finding is the direct detection of these compounds within the microenvironment of the breast. As previously stated, non-persistent hydrophilic pesticides and their excreted metabolites circulate transiently in blood plasma and have been detected in maternal breast milk [28]. Conversely, persistent lipophilic pesticides accumulate preferentially in the rich adipose stroma of the mammary gland, where they remain stored for years as an endogenous xenoestrogenic reservoir [29].

The verified presence of these environmental chemicals within breast adipose tissue, blood serum, and breast milk confirms that these compounds may act directly on the vascularized stroma and epithelial cells of the mammary gland. This continuous, local, molecular-level exposure, whether through persistent adipose bioaccumulation or chronic plasma flux, provides a plausible mechanism for chronic endocrine disruption, cellular atypia, and the initiation and progression of breast cancer.

## 4. Pesticides and Their Role on the Development of Breast Cancer

To understand how environmental contaminants may influence the complex biology of the mammary gland, it is necessary to establish a clear toxicological framework regarding how they can interact with human physiology.

Exposure to pesticides activates adaptive cellular defense pathways, including xenobiotic-sensing nuclear receptors such as pregnane X receptor (PXR) and oxidative stress-responsive pathways centered on nuclear factor erythroid 2-related factor 2 (Nrf2), thereby regulating detoxification enzymes and antioxidant defenses [30,31]. While initially cytoprotective, the chronic activation of these pathways upregulates the ATP-binding cassette (ABC) transporter superfamily, specifically the multidrug-resistance gene 1-type/p-glycoprotein (MDR1/P-gp) and the breast cancer resistance protein (BCRP). These active efflux pumps purge cytotoxic chemotherapeutic drugs, such as paclitaxel and doxorubicin, out of the cell, rendering standard treatments ineffective [32]. Simultaneously, the pesticides expand the highly resilient clusters of differentiation (CD)44+ and CD24− cancer stem cell niche and suppress the mitochondrial apoptotic cascade [33]. This establishes a highly aggressive, drug-resistant cellular reservoir capable of surviving conventional chemotherapy and radiotherapy, ultimately leading to clinical recurrence and metastatic progression. While pesticides are designed to disrupt biochemical pathways unique to target organisms, such as the nervous system of insects or the metabolic machinery of plants, their biological activity is rarely species-specific. Upon entering mammalian systems, these compounds frequently interact with highly conserved cellular targets, triggering unintended systemic pathologies [23,34]. Due to their chemical structures, often featuring aromatic rings and specific functional groups, many pesticides exhibit “molecular mimicry,” structurally resembling endogenous steroid hormones, particularly 17β-estradiol (E2) [35]. This structural similarity allows them to bypass the body’s strict homeostatic feedback loops and disrupt endocrine signaling through three primary avenues: receptor agonism and antagonism, enzymatic modulation and epigenetic priming. In particular, many pesticides may bind directly to ERs, and GPER [36]. This binding can trigger inappropriate oncogenic gene expression or block natural ligands, disrupting cellular homeostasis [36,37]. In addition, EDCs can directly interfere with steroidogenesis. For example, several herbicides and fungicides upregulate the aromatase activity which converts androgens into estrogens, thereby driving local hyper-estrogenism within the breast microenvironment [38]. Finally, exposure to these compounds can alter DNA methylation patterns and histone modifications. These alterations permanently rewrite cell differentiation pathways in the mammary tissue without changing the genetic sequence itself [39].

Because the mammary gland is an exquisitely hormone-sensitive organ whose development, cyclical remodeling, and lactation are guided by a precise orchestration of estrogens, progesterone, and prolactin, it is highly vulnerable to EDC-induced disruption. Even at environmentally relevant concentrations within the nanomolar to picomolar (pM) ranges, reflecting internal bioaccumulation levels reported in human serum and adipose tissue biopsies, chronic exposure to hormone-mimicking pesticide mixtures can stimulate cellular proliferation, interfere with normal tissue, and predispose healthy epithelial cells to future neoplastic transformation.

Depending on their intended agricultural targets and chemical structures, pesticides discussed in this review are classified into three main functional categories: herbicides (organophosphonates and triazines, such as glyphosate and atrazine, respectively), insecticides (organophosphates and legacy organochlorines, such as chlorpyrifos and p,p′-DDT and its primary persistent metabolite p,p′-DDE, respectively), and fungicides (azoles and dithiocarbamates, such as fenhexamid and mancozeb, respectively).

### 4.1. Herbicides: Glyphosate and Atrazine

Synthetic herbicides represent the most widely applied agrochemicals globally [25,40]. Among these, glyphosate [N-(phosphonomethyl) glycine], an organophosphorus herbicide targeting the 5-enolpyruvylshikimate-3-phosphate synthase enzyme in plants [40], has been subject to extensive toxicological evaluation and regulatory scrutiny [41,42]. In 2015, the International Agency for Research on Cancer (IARC) categorized glyphosate as “probably carcinogenic to humans” (Group 2A), citing sufficient evidence in experimental animals, limited epidemiological evidence in humans (primarily for non-Hodgkin lymphoma), and strong mechanistic indicators of genotoxicity and oxidative stress [42,43,44,45,46,47]. However, other major regulatory bodies, including the European Food Safety Authority (EFSA) and the US Environmental Protection Agency (EPA), concluded that current evidence does not support a direct carcinogenic classification for technical-grade glyphosate.

In the context of breast oncology, evidence regarding glyphosate remains predominantly preclinical and mechanistic [48]. In vitro studies utilizing human ER-positive breast cancer cell lines (e.g., MCF-7) demonstrate that pure glyphosate exhibits weak xenoestrogenic activity [49]. Specifically, in vitro assays show that nanomolar concentrations of pure glyphosate weakly stimulate the transcriptional activity of the subtype alpha of ER (ERα) both through direct ligand-binding domain interactions and ligand-independent phosphorylation pathways [49]. Concurrently, in vitro human cell line experiments report a modest upregulation of the cytochrome P450 family 19 subfamily A member 1 (CYP19A1) expression, suggesting a potential modulation of local steroidogenesis [38]. Furthermore, in vivo rodent exposure models evaluating multigenerational GBH administration suggest an epigenetic transgenerational inheritance (F1–F3) of mammary tumor susceptibility [50].

Regarding metabolic and transgenerational pathways, in vitro human cell models show that the primary environmental metabolite aminomethylphosphonic acid (AMPA) independently induces oxidative stress and cell cycle alterations [51]. In contrast, a prospective human epidemiological biomonitoring study reported a positive statistical association between elevated urinary AMPA excretion and postmenopausal breast cancer risk [52].

Crucially, a clear distinction must be drawn between pure glyphosate and commercial glyphosate-based herbicides (GBHs) [53,54]. Comparative in vitro testing on human cell lines demonstrates that commercial GBH formulations, which contain patented surfactants and adjuvants such as polyethoxylated tallowamine, exert significantly higher cytotoxicity, membrane permeabilization, and reactive oxygen species (ROS) generation than the active ingredient alone [53,54,55]. While sustained ROS generation in human mammary cell lines can lead to lipid peroxidation and transient DNA strand breaks [41,44], these pro-oxidant effects are predominantly driven by formulation additives rather than pure glyphosate [54,55].

Although glyphosate is the main organophosphonate-based product used for non-selective weed control, the agronomic management of broadleaf and grass weeds has historically relied on selective chemicals, in particular triazine-based herbicides [56,57]. In contrast to glyphosate inhibition of aromatic amino acid synthesis, triazine derivatives exert their phytotoxicity by targeting the photosynthetic apparatus. Specifically, triazines bind competitively to the QB-binding niche on the D1 protein within photosystem II (PSII), blocking photosynthetic electron transport and inducing oxidative damage in plant cells [56,57]. Unlike glyphosate, which is rapidly absorbed by soil matrices, triazines have a significantly greater potential to leach into groundwater and surface water [58]. Decades of intensive application have driven the evolution of target-site resistance across numerous weed species, prompting increasing regulatory restrictions worldwide [59,60].

In this context, while some population-based epidemiological studies initially explored potential links between triazine herbicide usage and breast cancer incidence in agricultural basins [61], cumulative epidemiological evidence remains inconclusive and inconsistent [61,62]. Major international evaluation bodies, including the IARC (Group 3: not classifiable as to its carcinogenicity to humans) and the US EPA, have concluded that human data are inadequate to establish a causal relationship between atrazine exposure and elevated breast cancer risk [61].

A critical factor in evaluating atrazine toxicity is the sensitivity of the species. In vivo studies using Sprague-Dawley rats demonstrate that atrazine disrupts the central neuroendocrine axis, blocking the pre-ovulatory luteinizing hormone (LH) surge and forcing a state of persistent estrus coupled with hyperprolactinemia, which promotes mammary tumor development in aging rats [63]. However, this specific neuroendocrine sensitivity and reproductive senescence sequence are characteristic of rodent biology and do not directly map onto human reproductive physiology or menstrual cycle regulation [64].

Mechanistically, preclinical in vitro experiments using human adrenocortical carcinoma and breast cancer cells show that atrazine does not act as a direct agonist for classical nuclear ERs [63,65]. Instead, in vitro assays in human mammary epithelial cells and stromal fibroblasts indicate that atrazine can act as an indirect endocrine disruptor by stimulating intracellular cyclic adenosine mono-phosphate/protein kinase A (cAMP/PKA) signaling, which upregulates the transcription of the CYP19A1 gene and increases local aromatase activity [61,63,64,65]. Furthermore, in vitro human cell models demonstrate that atrazine can induce rapid non-genomic signaling through GPER transactivation of epidermal growth factor receptor (EGFR) and the downstream mitogen-activated protein kinase/extracellular signal-regulated kinase (MAPK/ERK) cascade [66]. Overall, while atrazine exhibits well-documented aromatase-inducing and GPER-activating capabilities in experimental cell models, current epidemiological and toxicological data do not support an established causal link between real-world atrazine exposure and human breast cancer [61,62,64,67].

### 4.2. Insecticides: Chlorpyrifos and p,p′-DDT/p,p′-DDE

The molecular alterations induced by herbicides, particularly regarding genomic instability and mitochondrial dysfunction, share critical convergence pathways with organophosphate insecticides [68]. Among these, chlorpyrifos represents another pervasive environmental threat to mammary tissue homeostasis [69]. Epidemiological and biomonitoring studies have established a concerning link between chlorpyrifos exposure and an elevated incidence of breast carcinoma, particularly in premenopausal women [69]. While chlorpyrifos is primarily recognized for its neurotoxic profile via acetylcholinesterase inhibition [70], emerging data obtained in in vitro studies, using human breast cancer cell lines demonstrate that it promotes pro-metastatic traits and therapeutic resistance by inducing severe oxidative stress, driving epithelial–mesenchymal transition (EMT), and promoting resistance to standard oncological treatments [71,72]. At the cellular level, in vitro studies demonstrate that chlorpyrifos acts as a multi-modal endocrine disruptor. Although it behaves as a weak ERα agonist on its own, acute and long-term exposure triggers robust cell proliferation in both ERα-positive and triple-negative human breast cancer lines [71]. This proliferation is mediated not only by the cross-activation of ERα, but also through the hyperactivation of the aryl hydrocarbon receptor (AhR) and the upregulation of the cancer-associated enzyme neutral cholesterol ester hydrolase 1, namely KIAA1363 [73]. Furthermore, pesticide exposure triggers a significant intracellular redox imbalance. The resulting accumulation of hydrogen peroxide (H_2_O_2_) and ROS drives the sustained phosphorylation of ERK1/2 pathway, converting baseline oxidative stress into a persistent mitogenic signal [74]. Moreover, ROS activates pro-inflammatory transcription factors, most notably the nuclear factor kappa B (NF-kB) that directly upregulates the transcription, translation, and extracellular secretion of the matrix metalloproteinase (MMP)-2 and MMP-9 [71,72]. By digesting Type IV collagen and laminin within the basement membrane, these MMPs compromise the structural scaffolding of the mammary gland. This allows invasive breast cancer cells to break through local tissue barriers and enter the lymphatic and vascular systems [75]. More alarmingly, studies published in 2026 demonstrate that co-exposure to chlorpyrifos and tamoxifen alters the phenotypic plasticity of breast cancer cells, driving a “stemness” profile [76]. This selected cell subpopulation exhibits enhanced mammosphere-forming ability and a marked upregulation of core stemness markers such as CD44, the homeobox domain transcription factor (NANOG), and POU domain, class 5, transcription factor 1 (POU5F1), alongside a downregulation of CD24 [76]. This shift toward an undifferentiated, basal-like phenotype is orchestrated via the epigenetic regulation of histone deacetylase 1 (HDAC1), closely mimicking the molecular signature observed in patients with acquired endocrine therapy resistance [76]. Consequently, organophosphate contamination represents a critical, modifiable environmental determinant that actively undermines the efficacy of first-line breast cancer therapeutics.

Even though current-use herbicides and organophosphates exert transient biochemical stress on mammary epithelial cells, they represent a significantly different toxicological paradigm compared to legacy organochlorine pesticides. Persistent organic pollutants, exemplified by p,p′-DDT and its highly stable primary metabolite p,p′-DDE, present a unique, lifetime oncogenic risk to human health [29]. While acute or early-life exposure to p,p′-DDT acts primarily through direct ERα activation as a classic xenoestrogen, its principal metabolic degradation product, p,p′-DDE, is characterized by extreme lipophilicity and multi-decadal sequestration within the lipid-rich stroma of the human mammary gland [77]. Cross-sectional biomonitoring studies confirm that peritumoral breast adipose tissue acts as a major endogenous repository, exhibiting significantly higher concentrations of p,p′-DDE than the adjacent tumor mass [77]. Crucially, during physiological lipolysis (e.g., pregnancy, lactation, or weight loss), stored p,p′-DDE is mobilized directly into adjacent epithelial compartments, driving paracrine anti-androgenic signaling, adipokine imbalance, and stromal remodeling that favor aggressive phenotypes [78,79].

Furthermore, in vivo studies conducted on MMTV-Neu transgenic mice have shown that the localized administration of p,p′-DDE into the mammary fat pad, significantly accelerates tumor growth and promotes the onset of aggressive and highly invasive tumor phenotypes [79]. Consequently, the localized accumulation of organochlorine compounds in breast adipose tissue shifts the paradigm of environmental carcinogenesis from a simple endocrine-mimicking effect to a determining factor, persistent and structurally embed, in tumor progression and the aggressiveness of the phenotype.

### 4.3. Fungicides: Fenhexamid and Mancozeb

Fungicides, widely applied agrochemicals used to control fungal diseases in fruit, vegetable, and wine production, represent a critical yet understudied class of EDCs. Epidemiological cohorts, including occupational evaluations of agricultural workers and their families, have indicated a potential risk of breast carcinoma associated with chronic exposure to specific systemic fungicides [80]. At the molecular level, fungicides disrupt mammary tissue homeostasis through dual hormonal and non-hormonal pathways. Mechanistically, in vitro experiments on human breast cancer cells show that azole and dicarboximide derivatives (such as fenhexamid) trigger estrogen receptor ERα signaling, activate the phosphoinositide 3-kinase (PI3K)/protein kinase B (Akt) axis, and perturb cytochrome P450 aromatase activity [81,82]. In addition to azoles, dithiocarbamate fungicides such as mancozeb, represent a significant environmental hazard due to their dual capacity for systemic endocrine disruption and severe redox imbalance. Biomonitoring and animal studies link chronic mancozeb (and its primary metabolite ethylenethiourea) exposure with altered gonadotropin secretion, impaired ovarian steroidogenesis, and increased rate of hormone-related tumors [83]. In human breast cancer lines, mancozeb acts as a potent pro-oxidant: it triggers mitochondrial membrane depolarization, depletes intracellular glutathione, and causes excessive generation of ROS. Analogously to insecticides, the fungicide-induced oxidative stress cascade activates downstream signaling involving the upregulation of MMP-2 and MMP-9 [78]. Concurrently, mancozeb perturbs estrogenic homeostasis by modulating estrogen receptor signaling, creating a permissive microenvironment that drives cell proliferation, ECM remodeling, and metastatic intravasation in hormone-dependent breast cancer models [84].

Table 1 summarizes the impact of pesticide classes on breast cancer.

### 4.4. Pesticide-Driven EMT, Tissue Invasion, and Metastatic Progression

While the initiation of breast cancer involves the acquisition of genomic and epigenetic alterations in healthy epithelial cells, the clinical lethality of the disease is determined by its progression towards an aggressive, invasive phenotype that allows cancer cells to escape the primary tumor site, enter systemic circulation, and colonize distant organs. Epidemiological and toxicological studies show that chronic low-dose exposure to agricultural pesticides does not merely trigger early carcinogenesis. On the contrary, these contaminants act as powerful promoters of tumor progression, remodeling the tumor microenvironment, altering intracellular signaling networks, and favoring the selection of drug-resistant populations, thereby increasing cellular aggressiveness and limiting the effectiveness of conventional therapies.

Similar to tumor onset, the molecular mechanisms driving this progression depend heavily on the specific physicochemical properties and chemical classes of the pesticide involved. As abovementioned, the organochlorines compounds, characterized by high environmental persistence and high lipophilicity, lead to significant tissue bioaccumulation in human adipose tissue, including the mammary fat pad. This persistent microenvironmental reservoir drives chronic, long-term non-genomic estrogenic signaling via GPER/MAPK cascades, maintaining a tonic stimulus for cell detachment and migration. Although organophosphates exhibit lower environmental persistence and minimal bioaccumulation compared to organochlorines, recurrent daily exposures nevertheless trigger acute and repeated waves of transient oxidative stress and kinase cascade activation, thereby sustaining the expression of invasive factors.

The critical gateway to this metastatic cascade is the EMT, which enables polarized, tightly adherent epithelial cells to assume a migratory mesenchymal phenotype. Emerging molecular evidence indicates that exposure to environmentally relevant concentrations, specifically nanomolar doses (1–100 nM) corresponding to internal exposure levels detected in human biomonitoring cohorts, strongly activates EMT in both hormone receptor-positive and triple negative breast cancer (TNBC) cell models [85,86]. At the molecular level, pesticides initiate EMT by inducing a “cadherin switch”, the transcriptional downregulation of E-cadherin accompanied by the reciprocal upregulation of N-cadherin and vimentin [87]. This cellular remodeling is driven by the activation of conserved developmental signaling pathways, such as the transforming growth factor beta (TGF-β)/small mother against decapentaplegic (Smad) axis, where TGF-β binding phosphorylates Smad2 and Smad3 to translocate a nuclear complex that directly upregulates core EMT-inducing transcription factors, including Snail, Slug, Twist, and zinc finger E-box binding homeobox 1 (ZEB1) [86].

Concurrently, persistent exposure to organochlorines and recurrent exposure to organophosphates can inhibit or bypass the destruction complex of β-catenin, allowing its accumulation, nuclear translocation, and binding to T-cell factor/lymphoid enhancer factor (TCF/LEF) transcription factors to drive EMT gene expression [72,88]. Simultaneously, non-genomic xenoestrogenic signaling via GPER rapidly activates the MAPK/ERK pathway to stabilize Snail and Slug against proteasomal degradation [89]. These pathways allow breast cancer cells to lose rigid cell-to-cell junctions, detach from the primary tumor mass, and gain physical motility. To complete intravasation into blood and lymphatic vessels, cells must break through the basement membrane and extracellular matrix (ECM), a process facilitated by the pesticide-induced upregulation, secretion, and activation of MMP-2 and MMP-9 that disrupt normal homeostatic tissue architecture [90,91].

## 5. Protective Potential of Bioactive Phytochemicals: Preclinical Insights and Translational Limitations

To mitigate pesticide-induced biochemical stress at the cellular level, specific dietary bioactive phytochemicals have been evaluated in experimental preclinical models for their capacity to reinforce defense systems against chronic oxidative stress, mitochondrial damage, and persistent genomic instability [92,93]. Polyphenols such as resveratrol, epigallocatechin gallate (EGCG), and curcumin function as direct scavengers of ROS, thereby blunting ROS-driven MAPK/ERK hyperactivation and downstream pro-inflammatory cascades. Furthermore, these compounds act as epigenetic modulators in experimental systems, influencing HDAC activity and DNA methyltransferases (DNMTs) to restore normal expression profiles of tumor suppressor genes aberrantly silenced by chronic EDC exposure [92].

At the metabolic level, organosulfur compounds derived from cruciferous vegetables, most notably sulforaphane and indole-3-carbinol, induce the nuclear translocation of Nrf2 in non-malignant tissue. Activated Nrf2 binds to antioxidant response elements and upregulates key Phase II detoxification enzymes, including glutathione S-transferases (GSTs), NAD(P)H:quinone oxidoreductase 1 (NQO1), and UDP-glucuronosyltransferases (UGTs). In preclinical assays, this accelerated enzymatic conjugation promotes the clearance and excretion of active pesticide residues and their toxic metabolites before they can induce irreparable DNA strand breaks or promote stemness expansion within the mammary epithelium. Additionally, specific dietary antioxidants exert anti-invasive actions by directly downregulating nuclear factor kappa B (NF-κB) transcription, thereby attenuating pesticide-stimulated MMP-2/MMP-9 secretion and preserving matrix integrity [92].

This metabolic enhancement extends to several other plant families: organosulfur derivatives from Alliaceae, such as garlic and onions (including diallyl disulfide and trisulfide), selectively modulate Phase I cytochrome P450 enzymes to prevent the metabolic bioactivation of pro-carcinogenic pesticides into reactive intermediates, while concurrently boosting Phase II GST activity to accelerate toxicant excretion [92]. Similarly, dietary flavones abundantly found in Apiaceae (celery and parsley), predominantly apigenin and luteolin, preserve cellular bioenergetics in vitro by protecting mitochondrial membrane potential from pesticide-induced depolarization and inhibiting the aberrant upregulation of ABC efflux transporters linked to chemoresistance [92]. Furthermore, polyphenolic compounds from Ericaceae and Rosaceae (berry fruits and pomegranates), particularly ellagitannins and their gut microbiota-derived urolithins, attenuate pesticide-induced genomic instability in experimental models by promoting high-fidelity DNA repair through the upregulation of nucleotide excision repair machinery [92]. Finally, citrus flavanones from Rutaceae, such as naringenin and hesperidin, reinforce endogenous antioxidant enzymes like superoxide dismutase and catalase while repressing NF-κB transcription, thereby blunting pesticide-stimulated MMP-2 and MMP-9 secretion [92]. A comprehensive summary of the main molecular targets and dietary sources of these bioactive phytochemicals is provided in Table 2.

Altogether, this orchestrated activation of Phase II conjugation, ROS neutralization, and anti-inflammatory signaling may accelerate the systemic clearance of active pesticide residues and their toxic metabolites before they can induce permanent DNA strand breaks or promote stemness expansion within the mammary epithelium [92]. However, a critical translational bottleneck exists regarding human application. In human physiology, low oral bioavailability, rapid hepatic first-pass metabolism, and extensive Phase II conjugation (sulfation and glucuronidation) severely limit the systemic and tissue-specific concentration of active aglycones within breast tissue [92,94]. Consequently, while dietary phytochemicals provide valuable molecular insights into cellular cytoprotection under controlled conditions, current preclinical evidence must not be misinterpreted as clinical proof that dietary choices or nutraceutical supplements can reverse, counteract, or neutralize pesticide-induced breast carcinogenesis in exposed human populations [21,94].

While experimental dietary strategies offer valuable mechanistic insights, true population-level risk reduction requires systemic public health interventions. Regulatory bodies worldwide must transition from evaluating pesticides as isolated compounds to calculating safety thresholds based on the “cocktail effect”, accounting for the cumulative and additive toxicity of multiple co-occurring chemical residues [21,94]. Implementing stricter maximum residue limits in commercial food commodities, expanding organic agricultural practices, and establishing non-spraying buffer zones around residential areas and schools represent critical public health measures to decrease the systemic pesticide body burden across populations and reduce the global incidence of environmentally driven breast malignancies.

## 6. Discussion

A central paradigm highlighted by this review is the dual capacity of pesticides to act as potent EDCs and systemic chemical stressors [24,30]. When evaluating these findings, it is essential to distinguish between the mechanistic evidence provided by preclinical models and the observational data from human populations. Whilst in vitro human cell lines and in vivo animal studies demonstrate clear molecular pathways, such as receptor activation, oxidative stress and matrix remodelling, epidemiological studies in humans primarily document statistical associations, which are inherently subject to confounding factors and complex co-exposures in the real world.

One of the main challenges in translating toxicological knowledge into a population-level risk assessment lies in the inconsistency of findings across epidemiological studies assessing pesticide exposure and the incidence of breast cancer [21,26]. These discrepancies do not necessarily reflect the absence of a biological effect, but rather stem from fundamental methodological complexities and factors linked to practical realities [26,28]. First, human exposure rarely involves isolated active ingredients; real-world scenarios entail chronic exposure to complex chemical mixtures (‘cocktail effect’) and commercial formulations with uncharacterized adjuvants, which can synergistically amplify toxicity [51,55]. Second, accurate exposure assessment remains critical: retrospective studies relying on questionnaires or residential proximity are vulnerable to recall bias, whereas prospective biomonitoring often relies on single-point sampling, which fails to capture decades of chronic exposure [26,77]. Third, the timing of exposure is a key determinant of carcinogenic susceptibility. Periods of susceptibility, such as prenatal development, puberty, pregnancy and breastfeeding, are characterised by active remodelling of the mammary gland, during which the tissues’ vulnerability to endocrine disruption is significantly greater than following exposure in adulthood [29,78]. Consequently, studies that assess exposure exclusively in adult cohorts without considering exposure windows in early life may fail to identify significant long-term risk associations [29,79]. Addressing these methodological gaps through prospective biomonitoring and the stratification of exposure windows is essential for reconciling observational clinical data with established preclinical mechanisms.

Bearing this distinction in mind, the human mammary gland, a structure that is highly sensitive to hormones and rich in specialised adipose stroma, clearly emerges as a primary bioaccumulative target for persistent lipophilic xenobiotics [29,36].

Legacy organochlorines like p,p′-DDT and its stable metabolite p,p′-DDE demonstrate the long-term oncogenic impact of tissue sequestration [29,77]. While p,p′-DDT exerts direct xenoestrogenic effects on epithelial cells during critical exposure windows, p,p′-DDE accumulates within peritumoral fat pads, converting the local adipose stroma into a persistent xenoestrogenic and anti-androgenic reservoir [77,78]. During lipolytic states, p,p′-DDE is mobilized directly into adjacent compartments, altering adipokine ratios (leptin/adiponectin) and fueling persistent low-grade tissue inflammation [78,79]. Contemporary herbicides like atrazine demonstrate that direct binding to classic nuclear receptors is not a prerequisite for endocrine disruption [61]. By stimulating intracellular cAMP/PKA signaling networks, atrazine transcriptionally upregulates CYP19A1 in both epithelial and stromal cells [61,63,64]. This local enzymatic hyper-induction forces the conversion of androgen precursors to active estrogens, establishing a self-sustaining, hyper-estrogenic microenvironment within the breast [61,64]. Coupled with non-genomic GPER/EGFR transactivation and systemic hypothalamic-pituitary-gonadal axis dysregulation, atrazine acts as an enzymatic engine driving sustained mitogenesis and genomic instability [61,65,67]. Beyond tumor initiation, pesticide exposures serve as critical drivers of malignant progression and clinical lethality by dismantling structural extracellular barriers [20,85,86]. Through ROS-mediated activation of pro-inflammatory cascades, organophosphates, organochlorines, and dithiocarbamates trigger the aberrant transcription and secretion of MMP-2 and MMP-9 [71,72,84,91], thereby facilitating EMT, basement membrane disruption, and subsequent metastatic intravasation into vascular and lymphatic networks [75,87,90].

It is crucial to emphasize that the molecular mechanisms discussed herein represent specific pathways identified for key chemical families rather than a universal feature of all agrochemicals. For instance, while legacy organochlorines rely on lipophilic sequestration in peritumoral fat pads to exert long-term paracrine xenoestrogenic effects, non-persistent water-soluble herbicides like atrazine act primarily via localized enzymatic hyper-induction of aromatase without long-term adipose bioaccumulation. These distinct toxicokinetic behaviors underscore why toxicological conclusions regarding tissue accumulation versus transient systemic exposure must remain strictly compound- and class-specific.

A critical caveat in interpreting these preclinical insights concerns the translational gap between experimental concentrations and realistic human internal exposure [21,26,28]. Large-scale human biomonitoring cohorts and adipose biopsy studies typically report internal pesticide burdens in the nanomolar to picomolar ranges [26,28,77]. However, a substantial body of published in vitro literature relies on micromolar concentrations to elicit detectable biological responses, such as acute ROS overproduction, MMP upregulation, or cell viability alterations [41,71,84]. These high-dose in vitro scenarios often reflect acute cytotoxicity rather than the chronic, low-dose, subtle endocrine disruption characteristic of real-world human exposure [24,35]. Conversely, certain non-genomic xenoestrogenic mechanisms operate effectively at environmentally relevant low concentrations [36,37]. Furthermore, localized tissue bioaccumulation must be considered: highly lipophilic persistent compounds sequester preferentially within the lipid-rich peritumoral fat pads, where local tissue concentrations within the breast microenvironment can become significantly higher over decades of lifetime exposure than circulating plasma levels [29,78]. Nevertheless, future toxicological research must prioritize testing environmentally representative concentration ranges and chronic exposure regimens to ensure that observed preclinical pathways accurately reflect human risk [21,35].

From a translational and public health perspective, the findings summarized in this review emphasize the urgent need to bridge individual nutraceutical interventions with systemic regulatory reform [21,92,94]. While dietary phytochemicals, such as resveratrol, sulforaphane, and EGCG, offer valuable cellular protection in preclinical models by scavenging ROS, modulating DNA repair, and inducing Nrf2-mediated Phase II detoxification enzymes [92,93], their translation to human clinical efficacy is severely constrained by low oral bioavailability, extensive first-pass metabolism, and limited tissue accumulation [92,94]. Consequently, dietary interventions and nutraceutical supplements should not be regarded as a substitute for clinical prevention, nor are they sufficient to counteract ongoing chemical exposure at a population level [21,92,94]. Current regulatory frameworks systematically fail to capture real-world human exposure scenarios because they evaluate pesticides primarily as isolated, pure active ingredients [21,53,94]. As demonstrated by commercial formulations containing functional adjuvants and surfactants that promote the penetration of active ingredients, as well as by the additive toxicity of environmental metabolites such as AMPA, chemical mixtures present in the environment exert cumulative mixture effects (‘cocktail effects’), through the sum of concentrations or independent action, which can significantly exceed the safety thresholds of the individual compounds [51,54,55,94].

**Figure 1:**
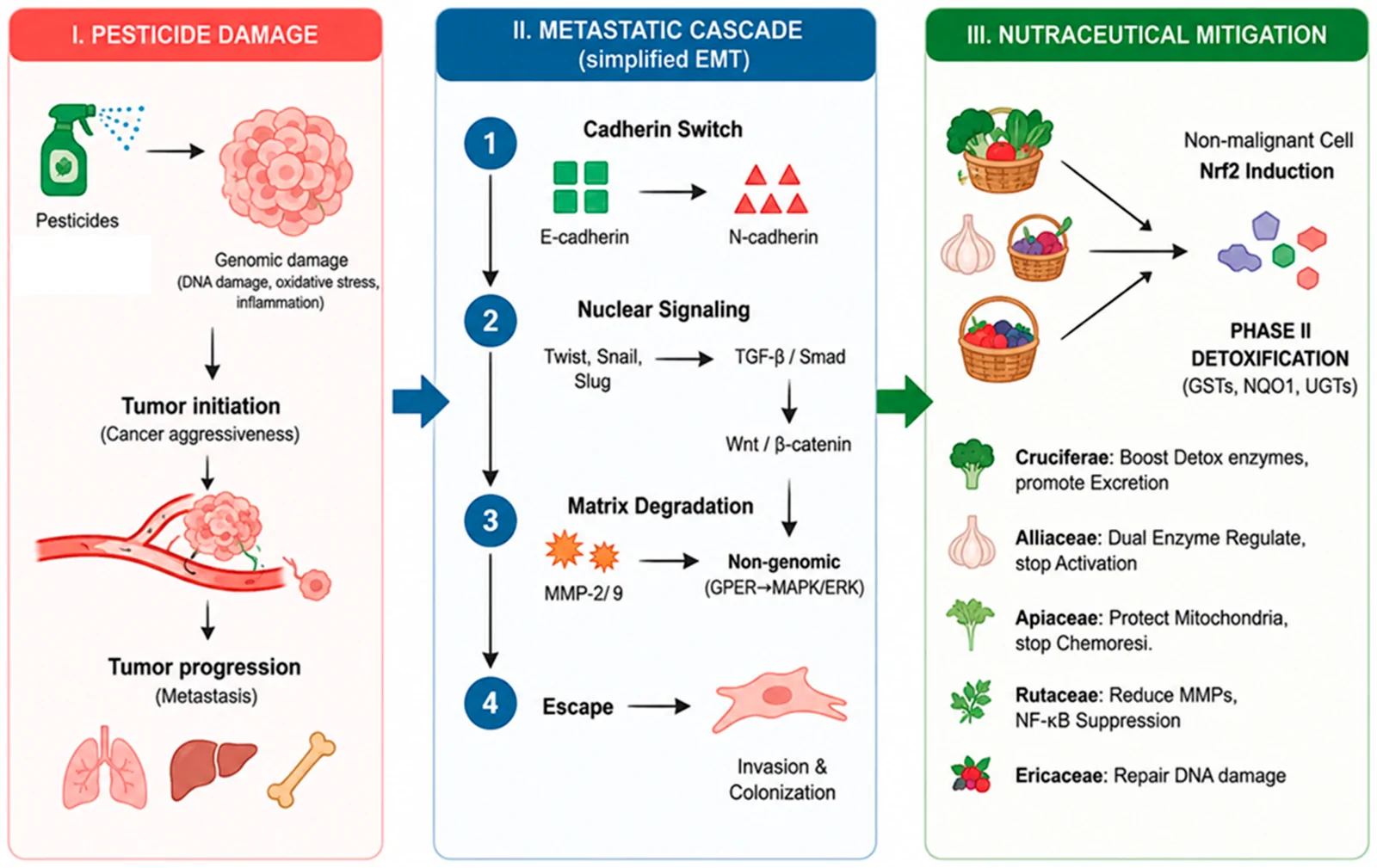
Molecular mechanisms of pesticide exposure in breast cancer progression and potential modulating roles of nutraceutic. Details are described in the main text. EMT: Epithelial–Mesenchymal Transition; TGF-β: Transforming growth factor beta; Smad: Small Mother Against Decapentaplegic; Wnt: Wingless-Related Integration Site; MMP: Matrix Metalloproteinases; GPER: G Protein-coupled Estrogen Receptor; MAPK: Mitogen-Activated Protein Kinase; ERK: Extracellular Signal-Regulated Kinase; Nrf2: Nuclear Factor Erythroid 2-related factor 2; GSTs: Glutathione S-Transferases; NQO1: NAD(P)H:Quinone Oxidoreductase 1; UGTs: UDP-Glucuronosyltransferases.

In conclusion, the integration of epidemiological, toxicological and molecular data will need to confirm the specific role of the various classes of pesticides as determining factors in both the onset of breast cancer and the aggressive progression of the disease [22,23,85,86]. Unraveling the precise molecular networks, ranging from the interaction between xenoestrogen receptors and the hyperinduction of aromatase to the activation of the EMT and the expansion of stem cell properties, could provide key targets for future primary prevention strategies and new therapeutic adjuvants [6,38,76,86]. Ultimately, reducing the overall burden of breast cancer requires a multi-tiered approach: deepening our molecular understanding of xenobiotic-induced carcinogenesis, establishing more rigorous public health policies that include assessments of the cumulative risk posed by chemical mixtures, and enforcing strict environmental regulations to reduce human exposure to endocrine-disrupting agrochemicals [21,24,94] (Figure 1).

## 7. Methodological Limitations and Knowledge Gaps

Despite these mechanistic insights, several methodological limitations and knowledge gaps must be acknowledged when translating experimental observations to human epidemiology. First, exposure misclassification remains a persistent challenge in observational cohorts; self-reported questionnaires are subject to recall bias, while single spot-sample biomonitoring (urinary or serum) fails to capture critical lifetime exposure windows or multi-decadal bioaccumulation dynamics. Second, observational human studies face complex confounding by lifestyle factors, reproductive history, body mass index, and dietary habits, which can obscure weak or moderate environmental signals. Third, significant discrepancies exist between experimental designs utilizing pure active substances and real-world human exposures to commercial formulations containing bioactive adjuvants and surfactants. Fourth, cross-sectional biomonitoring designs are vulnerable to reverse causation, as disease-induced weight loss, altered metabolic clearance, or cancer-associated lipid mobilization can artificially elevate circulating pesticide concentrations in patients. Finally, the vast majority of fine-grained molecular pathways remain validated strictly in vitro or in rodent models at concentrations exceeding typical human internal doses, underscoring the imperative for prospective cohorts incorporating longitudinal multi-omics biomonitoring.

## 8. Conclusions and Future Perspectives

In conclusion, the available epidemiological and experimental evidence supports legitimate concern regarding certain pesticide exposures in relation to breast cancer dynamics and identifies biologically plausible mechanisms, ranging from endocrine disruption and ROS-driven signaling to stromal remodeling, that warrant further investigation. While specific agrochemicals, including organochlorines, organophosphates, triazines, and dithiocarbamates, have been shown to interact with mammary tissue through distinct pathways, these findings must not be indiscriminately generalized to all pesticides or interpreted as definitive clinical proof of single-agent causality in humans. Future risk assessment and research strategies must account for the unique toxicokinetics, critical developmental exposure windows, and real-world cocktail dynamics of specific chemical formulations. By unraveling these class-specific molecular mechanisms and addressing current observational gaps, the scientific and medical communities can foster evidence-based environmental regulations and effective primary prevention strategies, ultimately mitigating the global burden of breast cancer.

## Figures and Tables

**Table 1:** Summary of the considered pesticide chemical classes, their molecular mechanisms, human epidemiological evidence levels, and observed biological impacts in breast cancer dynamics.

Pesticide Class	Main Molecular Targets and Mechanisms	Source/Model of Evidence	Observed Biological Impact	Level of Human Epidemiological Evidence and Evidence Tier	Main Toxicological/Oncogenic Role
Organophosphorus Herbicides (e.g., Glyphosate, AMPA)	ERα activation (direct binding and phosphorylation) CYP19A1 upregulation ROS overproduction, lipid peroxidation and DNA breaks Epigenetic alterations Commercial adjuvants enhance permeability	In vitro: Human ER+ and TNBC cell lines [38,40,41,42,43,44,45] In vivo: Rodent exposure models [46,47,48,49,50] Human Epidemiological: Biomonitoring cohorts [51,52,53,54,55]	In vitro: Proliferation, pro-inflammatory and survival pathways In vivo: Germline epimutations and transgenerational susceptibility (F1–F3) Epidemiological: Urinary AMPA correlates with postmenopausal risk	Preclinical, Emerging Biomonitoring/Epidemiological Data	Non-persistent enzymatic and epigenetic tumor promoter
Triazine Herbicides (e.g., Atrazine)	CYP19A1 hyper-induction via cAMP/PKA signaling Non-genomic GPER/EGFR transactivation (MAPK/ERK) HPG axis disruption (LH surge block, hyperprolactinemia)	In vitro: Human epithelial and adrenocortical cells [56,57,58,59,60] In vivo: Rodent models [61,62,63,64] Human Epidemiological: Agricultural cohorts [65,66,67]	In vitro: Mitogenic stimulation and phenotypic dedifferentiation In vivo: Persistent estrus and hyperprolactinemia Epidemiological: Elevated incidence in agricultural areas	Preclinical Mechanisms, Mixed/Regional Human Data	Local enzymatic driver of hyper-estrogenism
Organophosphate Insecticides (e.g., Chlorpyrifos)	Weak ERα agonist and AhR hyperactivation ROS-driven ERK1/2 activation NF-κB-driven MMP-2/9 secretion HDAC1-mediated epigenetic regulation	In vitro: Human ER+ and TNBC cell lines [68,69,70,71,72] Epidemiological: Agricultural Health Study cohorts [73,74,75,76]	In vitro: Enhanced migratory capacity, chemoresistance and CD44+/CD24− stemness expansion during tamoxifen exposure Epidemiological: Positive correlation with risk among healthcare workers’ partners	Preclinical Invasion Models, Observational Data	Acute/recurrent oxidative stressor and invasive promoter
Legacy Organochlorines (e.g., p,p′-DDT, p,p′-DDE)	p,p′-DDT: direct ERα activation (xenoestrogen) p,p′-DDE: Potent AR antagonism, peritumoral fat sequestration, mobilization during lipolysis Altered adipogenesis and adipokine ratio (leptin/adiponectin) Paracrine xenoestrogenic signaling	Human Biomonitoring: Patient fat pad biopsies [29,77] In vivo: MMTV-Neu mice [78] Epidemiological: Long-term prospective cohorts [29,79]	Biomonitoring: Stroma-driven metabolic plasticity and microenvironmental reprogramming In vivo: Accelerates kinetics and promotes aggressive TNBC/HER2+ phenotypes Epidemiological: Strong association following early-life exposure	Human Prospective and Biomonitoring Cohorts and Preclinical Data	Persistent, lipophilic microenvironmental promoter
Azole and Dicarboximide Fungicides (e.g., Fenhexamid)	Direct ERα activation PI3K/Akt survival pathway activation Perturbation of aromatase enzymatic activity	In vitro: Human breast cancer cell lines [80,81] Human Epidemiological: Occupational agricultural cohorts [82]	In vitro: Hormone-dependent cell survival and invasion Epidemiological: Potential elevated risk in female farmworkers	Preclinical Proof-of-Concept, Limited Direct Human Cohort Data	Endocrine disruptor and hormone-dependent mitogen
Dithiocarbamate Fungicides (e.g., Mancozeb)	Mitochondrial depolarization ROS-driven downstream MMP-2/9 upregulation Modulation of ERα and ovarian steroidogenesis	In vitro: Human breast cancer cell models [83] Epidemiological: Animal models and biomonitoring studies [78,84]	In vitro: Basal membrane disruption and invasive intravasation Epidemiological: Linked to endocrine disruption and hormone-related tumors	Preclinical Redox Stress, Emerging Observational Data	Redox disruptor and extracellular matrix remodeling factor

AMPA: aminomethylphosphonic acid; ERα: estrogen receptor alpha; CYP19A1: cytochrome P450 family 19 subfamily A member 1; ROS: Reactive Oxygen Species; TNBC: Triple Negative Breast Cancer; cAMP/PKA: cyclic Adenosine Mono-Phosphate/Protein Kinase A; GPER: G Protein-coupled Estrogen Receptor; EGFR: Epidermal Growth Factor Receptor; MAPK: Mitogen-Activated Protein Kinase; ERK: Extracellular Signal-Regulated Kinase; HPG: Hypothalamic-Pituitary-Gonadal axis; LH: Luteinizing Hormone; AhR: Aryl Hydrocarbon Receptor; NF-kB: Nuclear Factor kappa B; MMP-2/9: Matrix Metalloproteinases; 2 and 9; HDAC1: Histone Deacetylase 1; CD 44+/24−: clusters of differentiation 44 and 24; p,p′-DDT: Dichlorodiphenyltrichloroethan; p,p′-DDE: Dichlorodiphenyldichloroethylene; AR: Androgen receptor; MMTV-Neu: Mouse mammary tumor virus transgenic mice overexpressing NEU protein; HER2: Human Epidermal Growth Factor Receptor 2; PI3K/Akt: phosphoinositide 3-kinase/protein kinase B (Akt).

**Table 2:** Preclinical molecular mechanisms and translational bottlenecks of bioactive phytochemicals against pesticide-induced toxicity.

Compound Class	Major Dietary Sources	Primary Molecular Targets (In Vitro/In Vivo)	Translational/Bioavailability Bottlenecks
Polyphenols (Resveratrol, EGCG, Curcumin)	Grapes, Green Tea, Turmeric	ROS scavenging, MAPK/ERK suppression, HDAC/DNMT modulation	Rapid hepatic glucuronidation/sulfation; low aglycone concentration in target tissues [92,94]
Isothiocyanates (Sulforaphane)	Cruciferous vegetables	Nrf2 activation, induction of Phase II enzymes (GSTs, NQO1, UGTs)	High in vitro concentration requirements; short systemic elimination half-life [92]
Organosulfur derivatives (Diallyl sulfides)	Garlic, Onions	Modulation of Phase I CYP450, enhancement of Phase II GST clearance	High volatility; variable intestinal bioaccessibility and absorption [92]
Flavones and Flavanones (Apigenin, Naringenin)	Celery, Citrus fruits	NF-κB inhibition, MMP-2/9 downregulation, mitochondrial protection	Extensive intestinal and hepatic first-pass metabolic conversion [92,94]

EGCG: epigallocatechin gallate; ROS: Reactive Oxygen Species; MAPK: Mitogen-Activated Protein Kinase; ERK: Extracellular Signal-Regulated Kinase; HDAC: Histone Deacetylase; DNMT: DNA methyltransferases; Nrf2: nuclear factor erythroid 2-related factor 2; GST: glutathione S-transferases; NQO1: NAD(P)H:quinone oxidoreductase 1; UGTs: UDP-glucuronosyltransferases; CYP450: cytochrome P450; NF-κB: Nuclear Factor kappa B; MMP: Matrix Metalloproteinases.

## Data Availability

Data sharing is not applicable to this article. No new data were created or analyzed in this study.

## Abbreviations

The following abbreviations are used in this manuscript:

AhR	Aryl Hydrocarbon Receptor
Akt	Protein Kinase B
AMPA	Amino-methyl-phosphonic acid
BCRP	Breast Cancer Resistance Protein
BRCA	Tumor Suppressor Breast Cancer
cAMP	Cyclic Adenosine Mono-Phosphate
CD	Clusters of Differentiation
CYP19A1	Cytochrome P450 Family 19 Subfamily A Member 1 (aromatase)
DNMTs	DNA Methyltransferases
E2	17β-estradiol
EDCs	Endocrine-Disrupting Chemicals
EGCG	Epigallocatechin Gallate
EGFR	Epidermal Growth Factor Receptor
EMT	Epithelial–Mesenchymal Transition
ER	Estrogen Receptor
ERK	Extracellular Signal-Regulated Kinase
GBHs	Commercial glyphosate-based herbicides
GPER	G protein-coupled estrogen receptor
GSTs	Glutathione S-Transferases
H_2_O_2_	Hydrogen Peroxide
HDAC1	Histone Deacetylase 1
HER2	Human Epidermal Growth Factor Receptor 2
KIAA1363	Cholesterol Ester Hydrolase 1
MAPK	Mitogen-Activated Protein Kinase
MDR1/P-gp	Multidrug-Resistance Gene 1-Type/P-Glycoprotein
MeSH	Medical Subject Headings
MMP	Matrix Metal Proteinases
NANOG	Homeobox Domain Transcription Factor
ncRNA	non-coding RNA
NF-kB	Nuclear Factor Kappa B
NQO1	NAD(P)H:Quinone Oxidoreductase 1
Nrf2	Nuclear Factor Erythroid 2-Related Factor 2
p,p′-DDE	Dichlorodiphenyldichloroethylene
p,p′-DDT	Dichlorodiphenyltrichloroethane
PI3K	Phosphoinositide 3-Kinase
PKA	Activates Protein Kinase A
POU5F1	POU Domain, Class 5, Transcription Factor 1
PSII	Photosystem II
PXR	Pregnane X Receptor
ROS	Reactive Oxygen Species
Smad	Small Mother Against Decapentaplegic
TCF/LEF	T-Cell Factor/Lymphoid Enhancer Factor
TGF-β	Transforming Growth Factor Beta
TNBC	Triple Negative Breast Cancer
UGTs	UDP-Glucuronosyltransferases
ZEB1	Zinc finger E-box binding homeobox 1 (ZEB1)
